# BOL Lectin: A Protein Derived from Cauliflower Exhibits Antibiofilm Activity in In Vitro Assays Against *Staphylococcus aureus*

**DOI:** 10.3390/microorganisms13081901

**Published:** 2025-08-15

**Authors:** Leandro Augusto Mariano Silva, Natália Pereira, Mareliza Possa de Menezes, Romário Alves Rodrigues, Milena Souza Reis, Giordano Eugenio Oliveira, Hugo Leandro dos Santos, Lucas José Luduverio Pizauro, Ana Karen de Mendonça Ludgero, Christiane Eliza Motta Duarte, Leandro Licursi de Oliveira, Caio Roberto Soares Bragança, Marita Vedovelli Cardozo

**Affiliations:** 1 Department of Pathology Reproduction and One Health, School of Agricultural and Veterinary Sciences, São Paulo State University (UNESP), Jaboticabal 14884-900, Brazil; leandro.am.silva@unesp.br (L.A.M.S.); natalia.pereira1@unesp.br (N.P.); romario.a.rodrigues@unesp.br (R.A.R.); milena.s.reis@unesp.br (M.S.R.); 2 Department of Veterinary Clinic and Surgery, School of Agricultural and Veterinary Sciences, São Paulo State University (UNESP), Jaboticabal 14884-900, Brazil; mareliza.menezes@unesp.br; 3 Department of Biomedical Sciences and Health, Minas Gerais State University (UEMG), Passos 37900-004, Brazil; giordanoeoliveira@icloud.com (G.E.O.); anakludgero@gmail.com (A.K.d.M.L.); caio.braganca@uemg.br (C.R.S.B.); 4 Aquaculture Center, São Paulo State University (CAUNESP), Jaboticabal 14884-900, Brazil; hugo.leandro@unesp.br; 5 Department of Agricultural and Environmental Sciences, Santa Cruz State University (UESC), Ilhéus 45662-900, Brazil; ljlpizauro@uesc.br; 6 Department of General Biology, Federal University of Viçosa, Viçosa 36570-900, Brazil; leandro.licursi@ufv.br

**Keywords:** antimicrobial activity, biofilms, biotechnology, plant lectin, bacterial resistance, One Health

## Abstract

The BOL lectin, a 34 kDa protein with a hemagglutination titer of 64 hemagglutination units (HU), was extracted from cauliflower (*Brassica oleracea* spp. *botrytis* L.), purified by affinity and ion exchange chromatography, and confirmed, in this study, by Sodium Dodecyl Sulfate-Polyacrylamide Gel Electrophoresis (SDS-PAGE). The antibiofilm activity of BOL was evaluated at two concentrations (0.1 mg/mL and 1.0 mg/mL) against bacterial strains of importance to human health (*Bacillus cereus* ATCC 10876, *Escherichia coli* ATCC 25922, *Staphylococcus aureus* ATCC 29213, and *Streptococcus agalactiae* ATCC 12403). In addition to a biofilm formation assay, a pre-formed biofilm assay was conducted, with biofilm structure analyzed by Scanning Electron Microscopy (SEM). The antimicrobial potential of BOL was also investigated using the Minimum Inhibitory Concentration (MIC) assay in 96-well microplates. Among the tested bacterial strains, BOL exhibited activity against *S. aureus* at 1.0 mg/mL, interfering with both biofilm formation and disrupting pre-formed biofilms, which may be explained by a possible interaction between BOL and the components present in the biofilm matrix. However, no antibiofilm activity was observed against *E. coli*, *B. cereus*, or *S. agalactiae*, possibly due to differences in the composition of their biofilm matrices. Furthermore, BOL showed no detectable bactericidal or bacteriostatic activity in the antimicrobial assays. In conclusion, BOL lectin, at the tested concentrations, does not exhibit direct antimicrobial activity but effectively disrupts the extracellular matrix in *S. aureus* ATCC 29213.

## 1. Introduction

Lectins are proteins found in plants, microorganisms, fungi, and animals that, due to their carbohydrate-recognition domains, have the ability to bind to free carbohydrates or those attached to cell surfaces. This characteristic contributes to their involvement in various biological functions, including cell signaling, immunomodulatory activity, antimicrobial action, and others [1,2,3,4].

In the plant kingdom, several groups of lectins with unique domains have been identified, particularly in seeds, such as ricin, the first lectin isolated from castor bean extracts in 1888 [5]. In addition to seeds, lectins are also present in leaves, stems, roots, bark, flowers, and fruits [6].

Among the vast groups of plants within the plant kingdom, the Brassicaceae family stands out, comprising approximately 4.000 species of significant economic and scientific importance [7]. One of these species, *Brassica oleracea* spp. *botrytis* L., commonly known as cauliflower, was the subject of studies in 2017 aimed at isolating and purifying lectins. These efforts successfully led to the isolation and characterization of a novel plant lectin, named BOL (*Brassica oleracea* lectin) [8].

When first identified, the BOL lectin was distinguished by its conformational characteristics, attributed to the presence of characteristic MATH domains, as well as its stability within a pH range of 7.0 to 8.0 and tolerance to temperatures ranging from 4 °C to 60 °C [8]. Structural predictions revealed a β-sheet-rich architecture with two tandemly arranged MATH domains forming a β-sandwich fold, a configuration supported by circular dichroism analysis, which showed a typical spectrum for β-structured proteins with a pronounced negative band near 218 nm. Although BOL does not share sequence homology with classical lectins, its folding pattern and functional activity support its classification within this protein family [9]. Additionally, BOL demonstrated immunomodulatory activity, inducing phagocytosis and the production of inflammatory mediators by macrophages [8]. The presence of these immunomodulatory properties suggests that the protein may be effective against microbial pathogens, as lectins with antimicrobial activity interact with bacterial cell wall components, thereby inhibiting microbial growth. Furthermore, they affect membrane permeability, ultimately leading to microbial death [10]. Moreover, BOL may interact with structural and regulatory components of the biofilm, rather than acting solely on the bacterial cell itself, considering that Reference [8] reported the lectin’s ability to bind to complex sugars present in glycoprotein structures that are part of the extracellular matrix of certain bacteria.

From the perspective of One Health, there is growing concern regarding biofilm formation, which consists of bacterial communities that provide protection against external agents when exposed to unfavorable conditions. This process begins with bacterial adhesion, mediated by flagella, fimbriae, and pili, to a variety of surfaces, whether abiotic, such as steel tubing and drinking troughs, or biotic, such as skin and tissues. This associates biofilms with both human and animal infections [3,11,12].

Biofilms are more resistant to phagocytosis and antimicrobial agents than planktonic bacteria, as they interfere with drug action time and overall effectiveness [11,13,14]. Due to this resistance mechanism, there is increasing interest in the search for new therapeutic options, particularly of plant origin, that are capable of inhibiting both the growth of pathogenic microorganisms and biofilm formation [1].

Another important issue is antimicrobial resistance (AMR), a natural evolutionary mechanism that has been accelerated by the indiscriminate use of antimicrobials across various sectors, including human medicine, veterinary medicine, agriculture, and animal production [12]. AMR has become a global concern in recent years, with an increasing spread of drug-resistant strains to conventional treatments. If no effective and immediate measures are taken, AMR is projected to cause approximately 10 million deaths by 2050 [15].

For these reasons, this study reports the purification of *Brassica oleracea* spp. *botrytis* L. lectin and represents a pioneering effort to evaluate its antimicrobial and antibiofilm potential against major bacteria affecting human health.

## 2. Materials and Methods

### 2.1. Extraction and Purification of BOL Lectin

To obtain BOL lectin, the extraction and purification methods described by [8] were followed, with some modifications that did not compromise the tertiary structure or the biological activity of the BOL lectin. The cauliflower used was macerated in 50 mM Tris buffer, pH 7.4, at a 1:1 (*w*/*v*) ratio and left to rest under refrigeration for 8 h. The initial purification step of the crude extract was performed using a HiTrap Blue HP affinity column (GE Healthcare, Chicago, IL, USA) coupled to the NGC 10 Chromatography System (BIO-RAD^®^, Hercules, CA, USA). The column was pre-equilibrated with 50 mM Tris buffer, pH 7.4, and elution was carried out with 50 mM Tris buffer, pH 7.4/1 M NaCl. The fractions (PI) were collected using the NGC Fraction Collector (BIO-RAD^®^). Subsequently, dialysis was performed against 50 mM Tris buffer, pH 7.4, using Amicon^®^ Ultra Centrifugal Filters 30K (Merck^®^, Darmstadt, Germany), followed by a second pull-down purification using the Capto™ S ion exchange resin (Cytiva, Marlborough, MA, USA). The beads were equilibrated with 50 mM Tris buffer, pH 7.4, and incubated with the PI fraction for 1 h. Elution was performed with 50 mM Tris buffer, pH 7.4/100 mM NaCl. The obtained fraction (PII) was again subjected to dialysis against 50 mM Tris buffer, pH 7.4, using Amicon^®^ 30K filters (Merck^®^, Darmstadt, Germany). The fractions obtained during the purification steps, as well as the crude extract, were subjected to electrophoresis on a 12% polyacrylamide gel with sodium dodecyl sulfate (SDS-PAGE). After electrophoresis, the gel was stained with Coomassie Blue G-250 (Tokyo Chemical Industry, Tokyo, Japan) for 10 min and destained with a destaining solution. During all purification steps, the eluate fractions were monitored for hemagglutination activity to confirm the presence of the lectin.

### 2.2. Hemagglutination Assay

For the 96-well microplate assay, 50 µL of 0.9% saline solution was added in duplicate, followed by serial dilution of 50 µL of BOL lectin. Then, 25 µL of 2% rabbit erythrocytes, previously prepared in accordance with protocol number 21/2012, approved by the Animal Research Ethics Committee of the Federal University of Viçosa, was added to each well. The result of the assay was the highest dilution that exhibited hemagglutination, represented as the titer in hemagglutination units (HU).

### 2.3. Standardization of Bacterial Strains

The tests for evaluating antimicrobial and antibiofilm activity were conducted using Gram-positive bacterial strains such as *Bacillus cereus*, *Staphylococcus aureus*, and *Streptococcus agalactiae*, as well as the Gram-negative strain *Escherichia coli*, which were selected due to their clinical relevance and impact, as they are responsible for various infections and are also recognized as biofilm-forming organisms. Therefore, for the in vitro assays, the following standard strains were used: *Bacillus cereus* ATCC 10876, *Escherichia coli* ATCC 25922, *Staphylococcus aureus* ATCC 29213, and *Streptococcus agalactiae* ATCC 12403. The samples were stored in an ultrafreezer at −80 °C and were initially recovered in Brain Heart Infusion (BHI, Kasvi^®^) broth and incubated at 37 ± 2 °C for 24 h. Subsequently, the inocula were plated on Petri dishes containing Brain Heart Infusion (BHI, Kasvi^®^) agar and again incubated at 37 ± 2 °C for 24 h.

### 2.4. Biofilm Formation Assay

Interference with biofilm formation was evaluated as described by [16,17], with some modifications. Bacteria were inoculated in Tryptone Soy Agar (TSA) (Oxoid™, Hampshire, UK) supplemented with 2% glucose and incubated at 37 °C for 18 h; the colonies were then added to 2 mL of Tryptone Soy Broth (TSB) (Oxoid™) supplemented with 2% glucose and incubated again at 37 °C for 18 h. The cultures were then suspended with an automatic pipette in TSB broth supplemented with 2% glucose to standardize them to 10^8^ UFC/mL. Subsequently, 40 µL of each bacterial culture was dispensed in triplicate into the wells of a 96-well microtiter plate containing 40 µL of BOL lectin at concentrations of 50 and 1000 µg/mL, along with 20 µL of supplemented TSB. Ampicillin (32 µg/mL) was added as a positive control for *E. coli*, *S. aureus*, and *S. agalactiae* strains, and Vancomycin (32 µg/mL) for *B. cereus*. Such antimicrobials were selected based on the guidelines established by the Clinical and Laboratory Standards Institute (CLSI, Wayne, PA, USA) [18]. As a negative control, only supplemented TSB broth was added.

All experiments were performed in triplicate. The plates were incubated at 37 °C for 48 h, followed by aspiration of the contents, fixation with 99% methanol, washing with phosphate-buffered saline (PBS, pH 7.4), drying, and staining with 100 µL of 2% Crystal Violet for 1 min, followed by washing with distilled water. Biofilms were resuspended in 100 µL of 95% ethanol, and optical density (OD) was measured at 595 nm using an ELISA plate reader (Multiskan SkyHigh–ThermoFisher^®^, Thermo Fisher Scientific, Waltham, MA, USA).

### 2.5. Consolidated Biofilm Assay

The evaluation of the interference of BOL lectin in consolidated biofilm was performed as described by [16,19] with some modifications. The bacterial strains were seeded on Petri dishes with Tryptone Soy Agar (TSA) (Oxoid™) and incubated at 37 ± 2 °C for 24 h. Subsequently, the colonies were inoculated into 2 mL of TSB (Oxoid™) broth supplemented with 2% glucose and incubated at 37 °C for 18 h. The cultures were then suspended with an automatic pipette in TSB broth supplemented with 2% glucose to standardize them to 10^8^ UFC/mL. Subsequently, in 24-well microtiter plates containing coverslips previously prepared with poly-L-lysine, in triplicate, 300 µL of TSB (Oxoid™) supplemented with 2% glucose was added to each well, followed by the addition of 300 µL of the bacterial culture. The plates were incubated at 37 ± 2 °C for 48 h. The plates were then washed with 0.9% saline solution, and 100 µL of lectin was added to each well, followed by incubation at 37 ± 2 °C for 24 h. Ampicillin (32 µg/mL) was added as a positive control, and supplemented TSB broth was used as the negative control.

#### Scanning Electron Microscopy (SEM)

The coverslips with the already-formed biofilm were processed according to the routine protocol of the Electron Microscopy Center of the Institute of Biosciences at Botucatu (CME-IBB)–UNESP, Brazil. For this, the samples were fixed in 2.5% glutaraldehyde in 0.1M sodium phosphate buffer, pH 7.3, for at least 24 h, subjected to three washes in distilled water, and post-fixed in 0.5% osmium tetroxide in distilled water for 30 to 40 min. The samples were then washed three times in distilled water for 10 min and dehydrated in a graded series of alcohol. Next, the samples were subjected to critical point drying with CO_2_, fixed on stubs, and coated with gold. Finally, the materials were examined using a Quanta 200 Scanning Electron Microscope (FEI Company, Tokyo, Japan) located at the Electron Microscopy Center of the IBB, UNESP–Botucatu, São Paulo, Brazil.

### 2.6. Minimum Inhibitory Concentration (MIC) Assay

The minimum inhibitory concentration (MIC) of the BOL lectin was determined in triplicate using the standard broth microdilution technique established by the Clinical and Laboratory Standards Institute (CLSI) guidelines [18]. Bacterial suspensions were prepared in Cation-Adjusted Mueller Hinton Broth (CAMHB, Sigma-Aldrich^®^, Burlington, MA, USA) and adjusted to a 0.5 McFarland standard [18].

Next, serial dilutions of the lectin were prepared, ranging from 50 to 0.09 µg/mL and from 1000 to 1.95 µg/mL, in CAMHB broth (Sigma-Aldrich^®^), and added to 96-well microtiter plates. The previously prepared bacterial suspensions were then inoculated into the wells. Positive control wells, containing only the culture medium and bacterial strain, and negative control wells, containing only CAMHB, were also prepared to validate the test. After inoculation, the plates were incubated at 37 ± 2 °C for 18 h, and bacterial growth was evaluated. The MIC was defined as the lowest concentration of BOL lectin that inhibited visible growth in the wells.

### 2.7. Minimum Bactericidal Concentration (MBC) Assay

The MBC assay was performed using the protocol described by [20] with some modifications. Briefly, aliquots of 1 µL of bacterial suspension from the wells of the MIC microdilution plates, which exhibited variations in the BOL concentrations, were plated onto Brain Heart Infusion (BHI, Kasvi^®^, Izumi, Japan) agar plates and incubated at 37 ± 2 °C for 24 h. The MBC was defined as the lowest concentration at which no bacterial colonies were observed.

### 2.8. Statistical Analysis

The experiment was conducted using a completely randomized design. The absorbance variable was tested for normality and homoscedasticity using the Shapiro–Wilk and Levene tests, respectively. As the assumptions for parametric analysis were not fully met across all groups, non-parametric statistical methods were applied. The Kruskal–Wallis test was used to assess differences in biofilm formation across bacterial strains and treatments, and a significance level of 0.05 was adopted for all tests [21]. Analyses were performed using R software, version 4.0.5 [22].

## 3. Results

### 3.1. Protein Purification and Hemagglutination Assay

**Figure 1:**
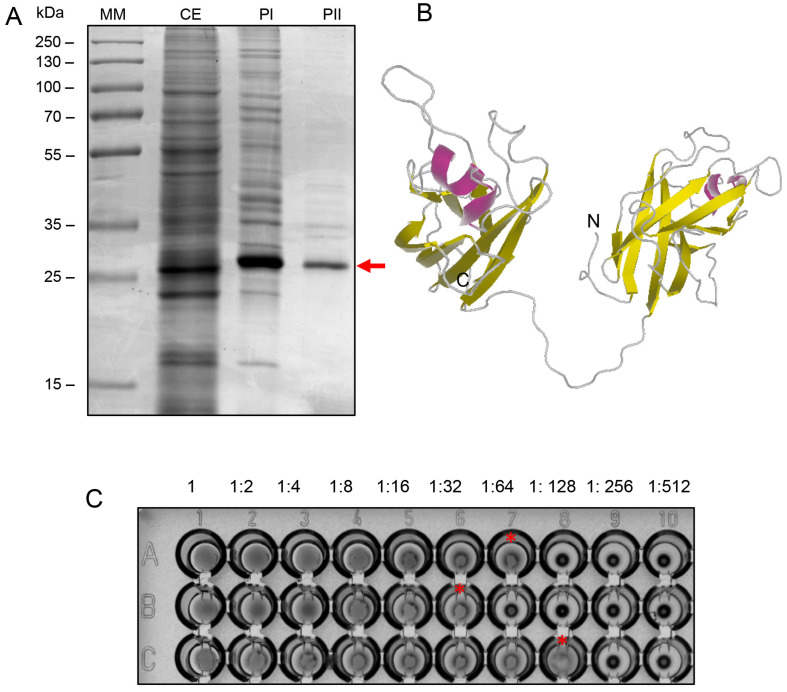
(**A**) SDS-PAGE (12% acrylamide), stained with Coomassie Blue R-250, showing the fractions collected during the chromatographic purification steps of BOL lectin. MM: molecular weight marker (Thermo Scientific™ PageRuler™ Plus Prestained Protein Ladder); CE: crude extract; PI: fraction obtained from affinity chromatography; PII: fraction obtained from ion exchange chromatography. The purified lectin, indicated by an arrow, is observed as a single band with an apparent molecular weight of 34 kDa. (**B**) Ribbon diagram of the predicted three-dimensional structure of BOL lectin. The helices are shown in pink, β-sheets in yellow, and loops in gray. The predicted structure reveals a model with 100% of the amino acid residues mapped into two tandemly arrayed MATH domains: domain 1 (residues 14–142) and domain 2 (residues 165–290). (**C**) Hemagglutination Assay. Hemagglutination activity was evaluated by serial twofold dilutions (from 1:2 to 1:512) of 50 µL of each sample with 25 µL of 2% rabbit erythrocyte suspension in PBS, in U-bottom microtiter plates. After 30 min of incubation at room temperature, agglutination was visually assessed. Panel A1–A10: crude extract; Panel B1–B10: affinity-purified fraction; Panel C1–C10: ion exchange-purified fraction. The endpoint titer was observed in 1:64 in the purified fraction (**C**), as indicated by the asterisk. The hemagglutination titer is defined as the highest dilution at which visible agglutination still occurs.

The BOL lectin was extracted and purified using a two-step chromatographic process. Figure 1A illustrates the electrophoretic profile of the crude extract (CE) and the fractions obtained after purification through a Blue Sepharose column (PI) and Capto S resin (PII). The SDS-PAGE gel analysis reveals that the lectin appears as a monomer with an approximate molecular weight of 34 kDa, consistent with the findings of [8]. The 3D structure of BOL was predicted using the RaptorX program (http://raptorx.uchicago.edu/, accessed on 8 April 2024), as depicted in Figure 1B [8]. The PII fraction exhibited an average hemagglutination titer of 64 HU, as shown in Figure 1C.

### 3.2. Biofilm Formation

**Figure 2:**
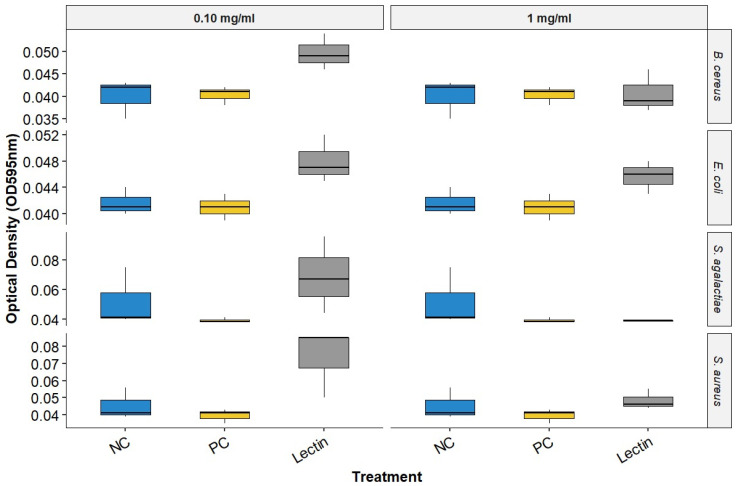
Biofilm Inhibition Assay of BOL. NC represents the supplemented TSB medium alone. PC indicates the bacterial strains treated with antimicrobials.

Upon evaluating the data on BOL’s interference with biofilm formation (Figure 2 and Table 1), it was observed that at a concentration of 0.1 mg/mL, BOL showed no significant difference (*p* > 0.05) compared to the other treatments. The protein was not effective in inhibiting biofilm formation, possibly due to its low concentration, which may have interfered with the interaction between the protein and the biofilm matrix. Additionally, its binding affinity may not have been strong enough to engage a sufficient number of glycan targets within the *S. aureus* biofilm matrix to produce measurable effects [8].

In the assay conducted with a concentration of 1.0 mg/mL, BOL did not produce statistically significant differences among treatments (*p* > 0.05). However, OD values in the BOL-treated groups were numerically like those observed for the positive control (PC), particularly for *Staphylococcus aureus*, suggesting a possible trend toward reduced biofilm formation compared to the negative control (NC). Although not conclusive, this pattern indicates that BOL may have the potential to modulate biofilm development in certain strains. This observation is further supported by scanning electron microscopy (SEM) images, which qualitatively revealed alterations in biofilm architecture following treatment with BOL at this concentration.

### 3.3. Consolidated Biofilm

**Figure 3:**
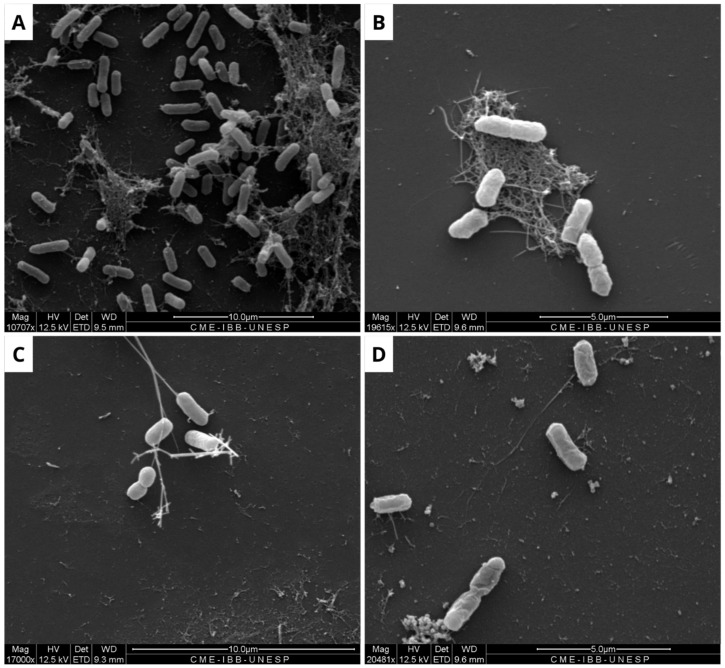
Scanning electron microscopy of *Escherichia coli* biofilm. The formation of untreated biofilm is observed in the negative control (**A**), while structural changes are evident in the treatment with Ampicillin (32 µg/mL) (**B**), BOL (0.1 µg/mL) (**C**), and BOL (1 µg/mL) (**D**). Scale bars range from 5 to 10 µm.

**Figure 4:**
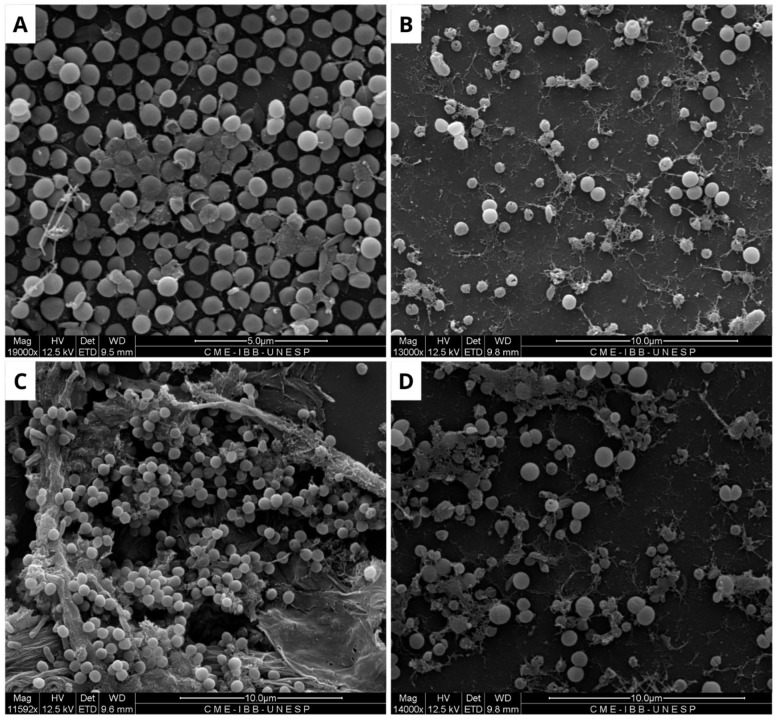
Scanning electron microscopy of *Staphylococcus aureus* biofilm. (**A**) Negative control, where biofilm formation is observed. (**B**) Positive control, showing modifications along the structure of the consolidated biofilm caused by Ampicillin (32 µg/mL). (**C**) Biofilm treated with BOL (0.1 µg/mL), showing no structural changes. (**D**) Treatment with BOL (1 µg/mL), showing loss of biofilm integrity. Scale bars range from 5 to 10 µm.

**Figure 5:**
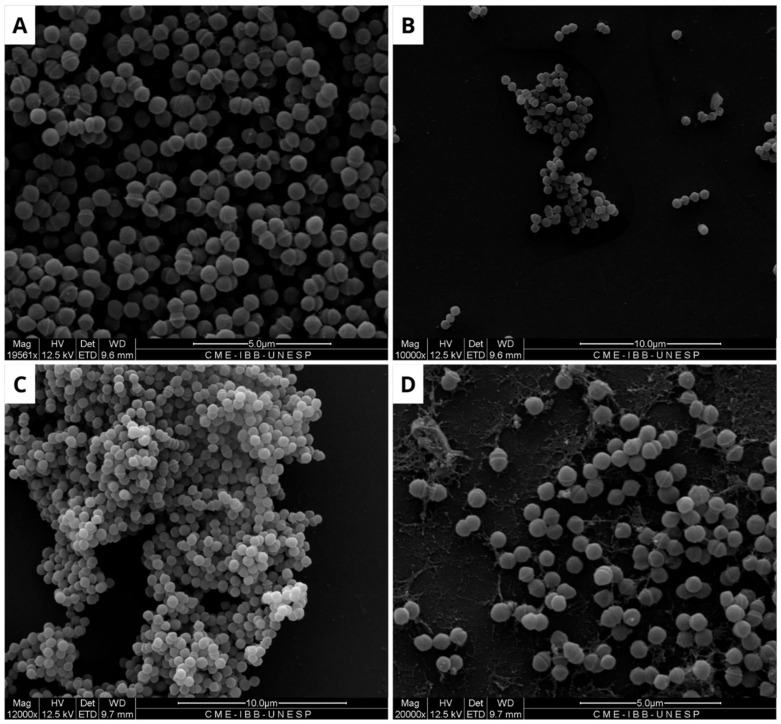
Scanning electron microscopy of *Streptococcus agalactiae* biofilm. (**A**) Negative control, showing biofilm formation in the absence of treatment. (**B**) Positive control, with structural modifications in the biofilm caused by Ampicillin (32 µg/mL). (**C**) Biofilm treated with BOL (0.1 µg/mL), showing no structural changes. (**D**) Treatment with BOL (1 µg/mL), showing loss of biofilm integrity. Scale bars range from 5 to 10 µm.

Due to the limited efficacy of BOL, its interference with pre-formed biofilms of *E. coli*, *S. aureus*, and *S. agalactiae* was evaluated. The results indicate that the protein interfered with the bacterial biofilm, with the most significant effect observed at a concentration of 1 mg/mL, as shown in Figure 3, Figure 4 and Figure 5. The structural effects of lectin on pre-formed biofilms were subsequently analyzed using scanning electron microscopy (SEM).

### 3.4. Minimum Inhibitory Concentration (MIC)

**Figure 6:**
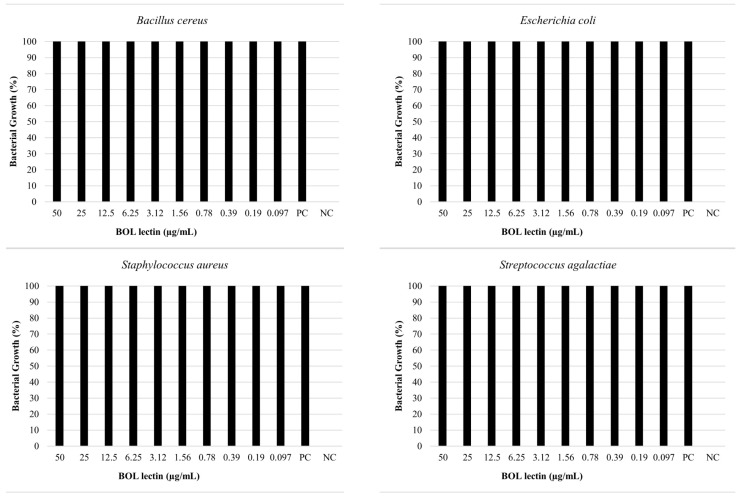
Effects of BOL lectin (0.1 mg/mL) on pathogenic bacteria. The positive control indicates only the bacterial inoculum and the culture medium, while the negative well represents the culture medium alone, serving as quality control for the test.

**Figure 7:**
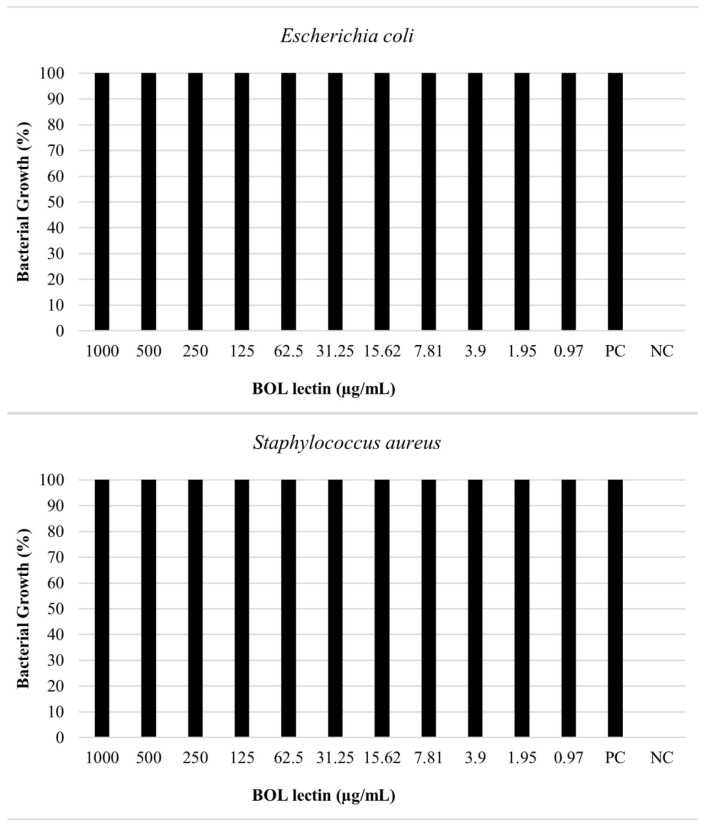
Effects of BOL lectin (1.0 mg/mL) on *Escherichia coli* and *Staphylococcus aureus*. The positive control indicates the well with only the bacterial inoculum and culture medium, while the negative well represents the culture medium alone, serving as a quality control for the test.

The antimicrobial assay of BOL lectin was performed using two different concentrations of the protein: 0.1 mg/mL (100 µg/mL) and 1 mg/mL (1000 µg/mL). The higher concentration (1 mg/mL) was tested only for *Escherichia coli* and *Staphylococcus aureus*, as these strains showed better results in the biofilm assays. However, neither concentration exhibited microbiostatic potential, as represented in Figure 6 and Figure 7. Furthermore, the minimum bactericidal concentration (MBC) assay demonstrated that all concentrations plated on Brain Heart Infusion (BHI, Kasvi^®^) agar showed growth, indicating the absence of bactericidal activity for BOL at the two evaluated concentrations.

## 4. Discussion

In this study, the lectin BOL, derived from *Brassica oleracea* spp. *botrytis* L., was evaluated for its antibiofilm and antimicrobial potential against the major pathogens encountered in human clinical settings, with an apparent molecular weight of 34 kDa, confirmed by SDS-PAGE. In previous work by [8], the identity and integrity of BOL were further validated by MALDI-TOF mass spectrometry and Edman degradation for peptide identification and sequencing. These analyses ensure the protein’s identity and reproducibility across studies. BOL was purified and tested for its ability to interfere with biofilm formation, as well as with pre-formed biofilm. The strains of *Bacillus cereus*, *Escherichia coli*, *Staphylococcus aureus*, and *Streptococcus agalactiae* were used in these assays.

Additionally, a hemagglutination assay was performed to validate the biological activity of BOL after its purification. Other hemagglutination inhibition assays were conducted by the group and described by [8], in which different types of monosaccharides and specific glycoproteins were evaluated. No inhibition of hemagglutinating activity was observed for any monosaccharides, but inhibition was observed with specific glycoproteins. These findings indicate that BOL recognizes complex glycan structures on glycoproteins rather than individual monosaccharides.

In general, the BOL lectin at its lowest concentration did not show significant efficacy in inhibiting biofilm formation of the evaluated strains, as no statistical differences were observed. This result is consistent with other studies that have also reported no significant effects when evaluating plant lectins in antibiofilm assays [1,23,24].

At a concentration of 1.0 mg/mL, although no statistically significant difference was observed, a reduction in absorbance (OD) values was noted in *S. aureus* when treated with BOL, with levels approaching those observed in the positive control (PC). While this trend does not confirm an antibiofilm effect, it suggests that BOL may interfere with biofilm formation in this strain. This potential activity could be related to interactions between BOL and bacterial surface components such as teichoic acid, peptidoglycan, or extracellular polysaccharides, which are known to play key roles in adhesion and biofilm structure. This finding corroborates the work of [3], which reported the ability of the plant lectins CrataBL and CrataBL-Lipo to inhibit *S. aureus* biofilm formation.

In contrast, regarding the structural evaluation of the consolidated biofilm conducted with SEM, the strains of *E. coli*, *S. aureus*, and *S. agalactiae* were assessed. It is noticeable, in general, that the biofilm formation pattern in the NC differs from that observed in the treatments with BOL and the antimicrobial (PC). This suggests that the lectin affected the composition of the bacterial biofilm, which is similar to findings reported by [25] when evaluating the WSMoL lectin. The effect of BOL on the matrix can be observed by the disruption of its structure. This action is likely due to the probable interaction of the lectin with various molecules that make up the cell surface, consequently affecting the process of biofilm matrix composition [26].

This is because lectins have a high specificity for carbohydrates due to their sugar-binding regions, which recognize specific structures based on precise stereochemical interactions. This characteristic allows different lectins to interact selectively with simple carbohydrates, such as monosaccharides, or more complex carbohydrates, such as oligosaccharides and glycoconjugates [27]. This specificity directly influences the antibiofilm activity of lectins, as the targeted recognition of glycoconjugates can compromise the formation and structural stability of the extracellular matrix of the biofilm [28]. In this context, the discrepancy between the absence of bactericidal or bacteriostatic activity and the effective disruption of biofilm structure by BOL lectin resembles the findings of [1,29], who reported biofilm modulation by lectins without affecting bacterial cell viability. This effect may be explained by the possible interaction of BOL with complex glycans present in the biofilm structure, mainly composed of EPS, rather than with cell surface receptors associated with bacterial growth or survival.

It is evident from the SEM images of *S. aureus* that BOL reduced biofilm formation, but this process was concentration-dependent. At the lower concentration of 0.1 mg/mL, the biofilm exhibited subtle alterations in its matrix, without complete disruption. In contrast, at a concentration of 1.0 mg/mL, the biofilm was significantly inhibited, with the rupture of the cell aggregates and the presence of matrix remnants. Although SEM analysis is qualitative, the structural changes observed in BOL-treated *S. aureus* biofilm at 1 mg/mL concentration are consistent with the OD reduction trend observed in the crystal violet assay. Similar characteristics were observed in the evaluation of the consolidated biofilm structure treated with the lectin jacalin associated with silver nanoparticles [30]. It is important to highlight that lectins can disrupt bacterial adhesion, reduce cellular viability, and modify the biofilm structure [31]. Thus, it is suggested that BOL is capable of disrupting the microbial community of *S. aureus*, due to a possible affinity of the protein for the components present in the structure that makes up the biofilm, particularly peptidoglycans (PGNs) located in the cell walls of Gram-positive bacteria. As reported by [32], *Staphylococcus aureus* exhibits relatively short-chain PGNs compared to *Bacillus subtilis*. Therefore, it is hypothesized that the structural characteristics of the bacterial cell wall are closely associated with BOL’s antibiofilm activity. Our study results are similar to the lectin isolated from the venom of the snake *Bothrops jararacussu* [29].

For *S. agalactiae*, in the SEM images of the BOL 0.1 mg/mL treatment, the presence of a cell aggregate is observed, which contrasts with all other images, including the NC. This fact indicates the activation of the bacterial defense mechanism in response to the proposed treatment, which provides the community with tolerance and resistance to the lectin. A similar result was obtained by [33], who evaluated the effect of the PgTeL lectin on biofilm formed by *Listeria monocytogenes* and observed the same behavior for the strain treated, which did not show any structural changes.

For the antimicrobial assay, our results indicated that the protein did not exhibit bactericidal or bacteriostatic effects against any of the bacteria tested, as 100% bacterial growth was observed at both concentrations (0.1 mg/mL and 1.0 mg/mL). This result is consistent with other studies, such as [34], which evaluated the antibacterial activity of the lectin Litchi, found in *Litchi chinensis*, using the disc diffusion method, and observed the absence of antibacterial potential against *Pseudomonas aeruginosa* at concentrations of 0.2 mg/mL and 0.4 mg/mL. Moreover, [1], when testing the Casul lectin found in leaves of *Calliandra surinamensis*, reported a bacterial inhibition potential of less than 50% for both Gram-positive and Gram-negative bacteria at a concentration of 0.1 mg/mL. These results suggest that some lectins, when evaluated in pure form or at low concentrations, may not exhibit antibacterial activity due to difficulty in targeting their action site, owing to the variability of carbohydrates found on bacterial cell surfaces [35].

Additionally, the inhibitory effect of a lectin may not be direct, as demonstrated by [36] in the expression of *Coprinopsis cinerea lectin 2* (CCL2) in *Arabidopsis*, which made the plants more resistant to fungal pathogens, including *Botrytis cinerea*, and the phytopathogenic bacterium *Pseudomonas syringae*. However, CCL2 was not able to inhibit the growth of *B. cinerea* in vitro assays. The authors propose that this lectin may act through the induction of defense-related genes.

However, there are studies that report the association of lectins with nanocarriers or antimicrobial agents, which significantly contributes to an increase in their efficacy. For example, CrataBL, when linked to nanocarriers, showed an improvement in antimicrobial activity. Similarly, DVL, when associated with antimicrobials, exhibited enhanced effects [3,34].

Additionally, like BOL, various plants have become targets for studies on the purification of proteins with potential medical applications, such as the lectin Afal, found in *Acacia farnesiana* seeds. This lectin was able to induce structural damage to the bacterial cell wall of *X. axonopodis* and *C. michiganensis* strains without affecting their metabolism [37].

In this context, the search for effective alternatives for the control and dissemination of antimicrobial resistance (AMR) is essential and should continue. Although BOL lectin did not show bacteriostatic effects in the tests performed, future research can be conducted to explore the association of BOL with an antimicrobial compound or nanocarrier. Furthermore, it is necessary to evaluate recombinant DNA techniques, as reported by [9], which can enhance the concentration of BOL lectin, efficiency, cost-effectiveness, and the viability of protein purification and application. Recombinant expression offers advantages such as scalability and batch-to-batch consistency, though challenges such as inclusion body formation, proper folding of lectins, and preservation of carbohydrate-binding activity must be carefully addressed [38].

While this study was based exclusively on Scanning Electron Microscopy (SEM), which provides qualitative analysis into biofilm architecture, future work should incorporate complementary quantitative approaches, such as biomass quantification or CFU enumeration. These analyses would enhance the robustness of findings and allow a more comprehensive evaluation of BOL’s antibiofilm potential.

## 5. Conclusions

In conclusion, although BOL lectin did not exhibit statistically significant antibiofilm activity under the conditions tested, a trend toward reduced biofilm formation was observed in *Staphylococcus aureus* at a concentration of 1.0 mg/mL, with OD values similar to those of the positive control. Additionally, scanning electron microscopy (SEM) revealed structural alterations in the biofilm matrix, supporting the hypothesis that BOL may interact with bacterial surface components. These findings suggest that BOL has potential as a biofilm-modulating agent, particularly for *S. aureus*, and encourage further investigation using additional strains, concentrations, and time points.

## Figures and Tables

**Table 1:** Mean optical density (OD595 ± SD) of biofilm formation by different bacterial strains treated with lectin (BOL), positive control (PC), and negative control (NC) at two concentrations (0.10 and 1.00 mg/mL).

Microorganism	Concentration	NC	PC	Lectin
*B. cereus*	0.10 mg/mL	0.040 ± 0.0044 ^Aa^	0.040 ± 0.0021 ^Aa^	0.050 ± 0.0040 ^Aa^
	1.00 mg/mL	0.040 ± 0.0044 ^Aa^	0.040 ± 0.0021 ^Aa^	0.041 ± 0.0047 ^Aa^
*E. coli*	0.10 mg/mL	0.042 ± 0.0021 ^Aa^	0.041 ± 0.0020 ^Aa^	0.048 ± 0.0036 ^Aa^
	1.00 mg/mL	0.042 ± 0.0021 ^Aa^	0.041 ± 0.0020 ^Aa^	0.046 ± 0.0025 ^Aa^
*S. agalactiae*	0.10 mg/mL	0.052 ± 0.0199 ^Aa^	0.039 ± 0.0017 ^Aa^	0.069 ± 0.0261 ^Aa^
	1.00 mg/mL	0.052 ± 0.0199 ^Aa^	0.039 ± 0.0017 ^Aa^	0.039 ± 0.0006 ^Aa^
*S. aureus*	0.10 mg/mL	0.045 ± 0.0093 ^Aa^	0.040 ± 0.0042 ^Aa^	0.073 ± 0.0202 ^Aa^
	1.00 mg/mL	0.045 ± 0.0093 ^Aa^	0.040 ± 0.0042 ^Aa^	0.048 ± 0.0059 ^Aa^

Different lowercase letters in a row indicate significant differences between treatments for a given microorganism (Kruskal–Wallis, *p* < 0.05). Different uppercase letters in a column indicate significant differences between microorganisms within treatment and concentration (Kruskal–Wallis, *p* < 0.05).

## Data Availability

The original data presented in the study are openly available in the UNESP Institucional Repository, at (https://hdl.handle.net/11449/295591, accessed on 20 June 2025). Further inquiries can be directed to the corresponding authors.
